# ATG3 is subjected to redox regulation to quarantee ATG8 lipidation under ROS-generating stresses

**DOI:** 10.1080/27694127.2023.2300622

**Published:** 2024-01-08

**Authors:** Manuel J. Mallén-Ponce, María Esther Pérez-Pérez

**Affiliations:** Instituto de Bioquímica Vegetal y Fotosíntesis, Consejo Superior de Investigaciones Científicas (CSIC)-Universidad de Sevilla Avda. Américo Vespucio 49, 41092 Sevilla, Spain

**Keywords:** ATG3, ATG8 lipidation, Chlamydomonas, ROS, redox, stress, Trx, yeast

## Abstract

The conjugation of ATG8 (autophagy-related 8) proteins to the lipid phosphatidylethanolamine (PE) is the result of the coordinated and highly regulated action of several ATG core proteins, including ATG4 proteases and the E1 (ATG7)- and E2 (ATG3)- activating enzymes. Although it has been stablished that ROS signaling plays an important role in autophagy activation, the molecular mechanisms underlying the redox control of ATG proteins remain largely unclear. We have recently shown that ATG3 activity in Chlamydomonas reinhardtii is subjected to reversible redox regulation to ensure ATG8 lipidation and autophagy progression under ROS-linked stress conditions.

**Abbreviations:** ATG, Autophagy-related; Cys, Cysteine; DTT_red_, Reduced Dithiothreitol; MM(PEG_24_), Methyl Polyethylene Glycol Maleimide; NEM: N-ethylmaleimide; PE, Phosphatidylethanolamine; ROS, Reactive Oxygen Species; TOR, Target Of Rapamycin; Trx, Thioredoxin; WT, Wild Type

Compelling evidence indicates that autophagy is upregulated in response to stresses to maintain cellular homeostasis and promote cell survival. In several of these unfavorable conditions, there is a pronounced increase of ROS production that may also act as secondary messengers during autophagy progression. In this regard, specific ATG proteins involved in the different steps of autophagy might be the direct targets of the redox regulation of this catabolic process. The lipidation of ATG8 is a key step in autophagosome biogenesis and essential for autophagy progression. The formation of the ATG8-PE depends on the regulation of ATG4, ATG7 and ATG3 activities. The presence of conserved Cys residues, including the catalytic Cys, might indicate that these three enzymes respond to redox signals, something already shown for ATG4 proteins.

The green microalga *Chlamydomonas reinhardtii* has recently emerged as a useful photosynthetic model organism for studying autophagy for the following reasons: 1) The *Chlamydomonas* genome contains a single copy of all *ATG* genes, in contrast to plants, where *ATG* genes are usually in multigenic families; 2) The development of robust and specific tools to monitor autophagy in this alga; 3) As in other single-cell organisms, simple experimental set-ups can be used to investigate autophagy under a wide range of stress conditions. Several studies in this microalga have shown that autophagy is inhibited by the TOR (Target Of Rapamycin) signaling pathway and activated by different stresses including oxidative damage, nutrient limitation or metal toxicity. In most of these conditions, an important correlation between ROS production, ATG8 lipidation and autophagy activation has been observed. However, the molecular mechanisms underlying the redox control of autophagy are not fully understood.

We have previously shown that yeast and *Chlamydomonas* ATG4 proteases are reversibly inhibited by ROS. In both organisms, ATG4 is regulated by the formation of a single disulfide bond according to the intracellular redox potential. This disulfide bond has a very low redox potential that can be reduced by thioredoxin. In addition, ATG4 has a second level of regulation by the formation of high molecular weight oligomers under oxidizing conditions. In close agreement, ATG4B from mammals was previously shown to be a direct target of ROS in nutrient-starved cells.

In a recent study [1], we have performed a comprehensive analysis to investigate the redox regulation of ATG3. To this aim, we have first *in vitro* characterized recombinant ATG3 proteins from *Chlamydomonas reinhardtii* and *Saccharomyces cerevisiae*. Our results revealed that the reduction of ATG3 leads to the monomerization of the protein, whereas the oxidation of this enzyme results in the detection of oxidized monomeric and dimeric forms. Both reduction and oxidation of ATG3 are fully reversible and involve its catalytic Cys. Moreover, this dithiol-disulfide exchange reaction depends on the redox potential and is controlled by thioredoxin (CrTrxh1) [1].

To investigate the implications of this redox post-translational modification on ATG3 activity, we set up a cell-free assay to analyze ATG8 lipidation in the presence of redox agents. We mixed total extracts from wild-type (WT) *Chlamydomonas* cells with recombinant his-tagged CrATG3 under reducing (DTT_red_) or oxidizing (H_2_O_2_) conditions, followed by western blot with anti-CrATG8. With this assay, we could easily distinguish between free and lipidated CrATG8. We found that CrATG3 requires reducing equivalents to be active since no CrATG8 lipidation was detected in the absence of DTT_red_ [1]. These results were further confirmed using a similar assay with WT and *atg3Δ* yeast strains, and recombinant CrATG8 as substrate. We found that endogenous yeast ScATG3 is able to lipidate CrATG8 in DTT_red_-treated total extracts from WT cells [1]. Furthermore, the addition of recombinant ScATG3 to total cell extracts from *atg3Δ*cells allowed CrATG8 lipidation in the presence of DTT_red_ [1]. These *in vitro* and cell-free assays might also be used to characterize ATG3 proteins from other organisms.

Our final goal was to determine whether the redox state of ATG3 regulates ATG8 lipidation *in vivo*. To this purpose, we analyzed CrATG3 oxidation/reduction and CrATG8 lipidation in *Chlamydomonas* cells subjected to four different ROS-generating stress conditions that induce chloroplast damage and trigger autophagy in this organism: treatment with norflurazon (herbicide that blocks carotenoids biosynthesis), cerulenin (fatty-acid synthesis inhibitor), methyl viologen (photosystem I electron acceptor), or high light stress. To examine the redox state of CrATG3, we conducted redox western-blot by combining an *in vivo* alkylating assay with the Cys-blocking agents N-ethylmaleimide (NEM) and methyl polyethylene glycol maleimide [MM(PEG_24_)] followed by an immunoblot analysis with CrATG3 antibodies. Briefly, 1) NEM binds to free SH or S^−^ groups, which irreversibly blocks reduced Cys; 2) Oxidized Cys are reduced by DTT_red_; 3) MM(PEG_24_) binds to newly reduced Cys (after DTT_red_ reduction), leading to an increase of 2.4 kDa in the CrATG3 molecular weight. Thus, reduced and oxidized CrATG3 can be distinguished easily by western-blot. Our results indicated not only that CrATG3 is targeted by ROS *in vivo* but also that the CrATG3 redox state dynamically changes under stress. CrATG3 was partially oxidized in untreated cells but chloroplast damage resulted in a profound modification of the CrATG3 redox state. ROS-linked stresses led first to a pronounced oxidation of CrATG3 followed by a complete reduction of the protein at the time of highest CrATG8-PE detection [1] (Figure 1). Therefore, we proposed that the redox regulation of CrATG3 during stress has two phases: 1) an initial stage where CrATG3 is first oxidized in response to higher ROS production; 2) a second step where CrATG3 is mainly reduced and active, likely due to the action of cellular antioxidant defense components such as thioredoxins, to perform ATG8 lipidation and facilitate autophagosome biogenesis and autophagy progression to eliminate the ROS-damaged cellular components (Figure 1).

**Figure 1.**
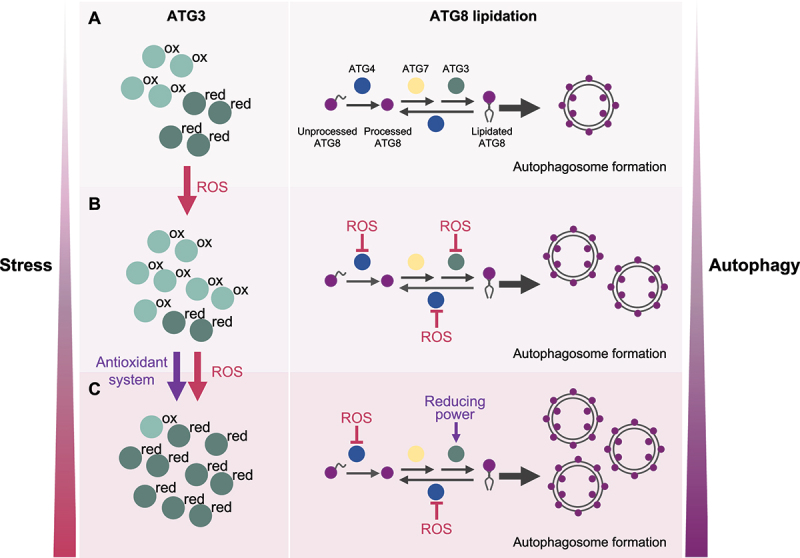
Model depicting the role of the ATG3 redox regulation in ATG8 lipidation.

Overall, our results highlight that the dynamic redox regulation of ATG3, a main component of the ATG8 lipidation system, controls autophagy activation under ROS-generating stresses. We propose that core ATG proteins, at least ATG4 and ATG3, respond to ROS signaling in a coordinated way to govern the balance between free ATG8 and lipidated ATG8 according to the environmental and intracellular cellular signals in Chlamydomonas cells (Figure 1). Whether these ATG proteins are subjected to other redox-based modifications such as nitrosylation or glutathionylation, or other post-translational modifications including phosphorylation remains unexplored.

We propose a model in which ATG3 can be found as reduced or oxidized forms depending on the redox cellular state. During optimal growth (A), ATG3 is partially oxidized and a low degree of ATG8 lipidation and basal autophagy are detected. Upon stress (B), the level of ROS increases and ATG3 is mainly oxidized and inhibited. In these conditions, ATG4 is also inhibited by ROS and ATG8 deconjugation is prevented. As result, ATG8 is lipidated and the formation of autophagosomes is upregulated. Under prolonged stress (C), ATG4 remains inhibited whereas ATG3 is primarily reduced likely due to the action of antioxidant systems, leading to higher ATG8-PE formation and further autophagy activation. This regulation of ATG3 must be coordinated with the redox control of ATG4. Therefore, the redox state of ATG3 and ATG4 dynamically changes in response to stress to guarantee a high level of ATG8 lipidation and autophagy progression.
